# ARIH2 regulates the proliferation, DNA damage and chemosensitivity of gastric cancer cells by reducing the stability of p21 via ubiquitination

**DOI:** 10.1038/s41419-022-04965-9

**Published:** 2022-06-22

**Authors:** Shengjun Geng, Wen Peng, Xue Wang, Xiaosong Hu, Hanghua Liang, Jianbing Hou, Feng Wang, Gaichao Zhao, Muhan Lü, Hongjuan Cui

**Affiliations:** 1 State Key Laboratory of Silkworm Genome Biology, Southwest University, 400716 Chongqing, China; 2 Cancer Center, Reproductive Medicine Center, Medical Research Institute, Southwest University, 400716 Chongqing, China; 3 Chongqing General Hospital, University of Chinese Academy of Sciences, 400014 Chongqing, China; 4 Department of Gastroenterology, the Affiliated Hospital of Southwest Medical University, 646000 Luzhou, China

**Keywords:** Gastric cancer, Gastric cancer

## Abstract

Ariadne homolog 2 (ARIH2) is a key member of the RING-between-RING (RBR) E3 ligase family, which is characterized by an RBR domain involved in the polyubiquitination process. However, the molecular mechanism and biological function of ARIH2 in the pathogenesis of gastric cancer remain unclear. In this paper, we found that high ARIH2 expression is correlated with poor prognosis in gastric cancer patients and that ARIH2 can significantly promote the proliferation of gastric cancer cells. The effect of ARIH2 knockdown on colony formation and tumorigenesis of gastric cancer cells was also shown both in vivo and in vitro. Further mechanistic investigations revealed that ARIH2 interacts with p21 and induces p21 ubiquitination, and that the K48 residue of ubiquitin and the K161 residue of p21 play key roles in ARIH2-mediated p21 ubiquitination. We identified ARIH2 as an E3 ligase of p21 by an in vitro ubiquitination assay. In addition, ARIH2 knockdown induced DNA damage, and then induced cell apoptosis and regulated the chemosensitivity of gastric cancer cells after combined treatment with 5-fluorouracil. Generally, our results indicated that ARIH2 promotes the proliferation of gastric cancer cells and regulates p21 expression. These data demonstrate the need to further evaluate the potential therapeutic implications of ARIH2 in gastric cancer.

## Introduction

Gastric cancer (GC), one of the most widespread global diseases, is characterized by recurring attacks, strong drug resistance and high invasion into the surrounding normal tissue and vascularization and is a serious threat to the health of people worldwide [1]. Due to the diversification of people’s dietary habits and increasing work pressure, GC patients show a trend of younger age. At present, early GC patients can be cured by surgery, while advanced GC patients need comprehensive treatment depending on the pathological type and clinical stage of GC, which includes surgical treatment, radiotherapy, chemotherapy and biological targeted therapy [2]. Currently, the poor understanding of the molecular mechanism of GC has become the key challenge in the fight against GC. Therefore, further exploration of the deep pathogenesis of GC is urgently needed to improve the survival rate of GC patients.

ARIH2 belongs to the Ariadne subfamily ligase of RBR E3 ligase. At present, ARIH2 has been studied for its regulatory role in many malignant cancers [3, 4]. Evidence has shown that ARIH2 plays an important role in the occurrence and development of acute myeloid leukemia, human non-small-cell lung cancer and other cancers [5, 6]. As a regulatory factor that plays an important role in the immune response, inflammatory diseases and skeletal muscle development, ARIH2 has attracted extensive research attention in recent years [7–9]. However, there have been no studies concerning the regulatory mechanism and biological function of ARIH2 in GC.

p21 (CDKN1A, also known as p21^WAF1/CIP1^), encoded by the CDKN1A gene, is a member of the CIP/KIP family [10, 11]. This molecule is involved in cell cycle arrest, DNA replication and repair, cell proliferation and differentiation, aging and apoptosis, and is closely related to tumor development [12–14]. p21 can play not only an anticancer role, but also a carcinogenic role, depending on the cell type, cell background, subcellular localization and post-translational modifications [15, 16]. The known E3 ligases related to p21 ubiquitination include RNF126, SKP2 and UHRF2, all of which play vital roles in human malignant tumors [17–19]. It has been reported that p21 inhibits cervical cancer, pancreatic cancer, bladder cancer, gallbladder cancer and other cancers [20]. Therefore, it is necessary to strictly monitor the expression of p21.

In this study, our results revealed that ARIH2 promotes the proliferation of GC cells. Mechanistically, we confirmed the protein interaction between ARIH2 and p21. ARIH2 regulated the p21 stability via ubiquitination and ARIH2 overexpression increased the ubiquitination of p21 in an E3 ligase activity dependent manner. Our study also identified the key sites of ubiquitin and p21 in ARIH2-mediated p21 ubiquitination. Moreover, our research showed that the level of DNA damage in GC cells was obviously increased after ARIH2 knockdown and that ARIH2 knockdown induced significant apoptosis of GC cells after combined treatment with 5-fluorouracil in vivo and in vitro. Taken together, our findings indicated that ARIH2 promotes the proliferation of GC cells and regulates the expression of p21 via ubiquitination. Our research provides insights into the pathogenesis of GC and identifies ARIH2 as a potential treatment target.

## Materials and methods

### Cell lines, drugs, reagents and antibodies

All human GC cell lines (BGC823, HGC27, MGC803, MKN45, and SGC7901), normal gastric cell line (GES-1) and human embryonic renal cell line 293FT were obtained from the American Type Culture Collection (ATCC, Beijing, China). All cell lines were tested mycoplasma-negative. MG132 and CHX were obtained from Sigma (Shanghai, China). Anti-ARIH2, anti-p21, anti-p27, anti-CDK1, anti-CDK2, anti-α-Tubulin, anti-HA, anti-SKP2, anti-RNF126 and anti-UHRF2 antibodies were purchased from Proteintech (Wuhan, China). Anti-MYC, anti-Flag, anti-phospho-ATR, anti-phospho-ATM, anti-ɣ-H2AX and anti-cleaved-caspase-3 antibodies were obtained from Cell Signaling Technology (Shanghai, China). Anti-Ki67 and anti-Bcl2 antibodies were purchased from BD Biosciences. All antibodies were diluted according to the manufacturer’s instructions.

### Transfection and infection experiments and plasmids

Small-hairpin shRNAs for ARIH2 and p21 and a negative control shRNA (shGFP) were obtained from Gene Pharma Co. Ltd. (Shanghai, China) and were inserted into the pLKO.1 vector [21]. The ubiquitination plasmid that contained an HA tag was purchased from Addgene (Beijing, China). The recombinant plasmids containing full-length human ARIH2 and p21 cDNA cloned into the PCDH-CMV-MCS-EF1-Hygro vector were purchased from Youbao Company (Changsha, China). The ubiquitin mutant plasmids (K6R, K11R, K27R, K29R, K33R, K48R, K63R) containing the HA tag and the p21 mutant plasmids (K16R, K75R, K141R, K154R, K161R, K163R) containing the MYC tag were also purchased from Youbao Company (Changsha, China). For transfection and infection experiments, the target plasmids and packaging plasmids were transfected into 293FT cells by using the transfection reagent Lipofectamine 2000 (Invitrogen, Carlsbad, CA, USA). Lentiviruses were collected 48 h later and used to infect GC cells twice, 24 h per infection. The infected cells were screened by treatment with puromycin for 36 h, and the surviving cells were frozen and stored in liquid nitrogen for subsequent experiments. All the primers for the shRNA sequences are given in Table 1.

**Table 1:** Primers of shRNA.

shARIH2#1-forward(5′-3′)	CCGGGCTGGATGTGTCTAGGAGATTCTCGAGAATCTCCTAGACACATCCAGCTTTTTG
shARIH2#1-reverse(5′-3′)	AATTCAAAAAGCTGGATGTGTCTAGGAGATTCTCGAGAATCTCCTAGACACATCCAGC
shARIH2#2-forward(5′-3′)	CCGGCGACTCTGAAACAGCCAACTACTCGAGTAGTTGGCTGTTTCAGAGTCGTTTTTG
shARIH2#2-reverse(5′-3′)	AATTCAAAAACGACTCTGAAACAGCCAACTACTCGAGTAGTTGGCTGTTTCAGAGTCG
shp21-forward(5′-3′)	CCGGGAGCGATGGAACTTCGACTTTCTCGAGAAAGTCGAAGTTCCATCGCTCTTTTTG
shp21-reverse(5′-3′)	AATTCAAAAAGAGCGATGGAACTTCGACTTTCTCGAGAAAGTCGAAGTTCCATCGCTC

### Immunohistochemistry staining

Paraffin-embedded tumors were cut into 5 mm thick slices, and then the paraffin sections were dewaxed and hydrated. Then, paraffin slices were placed in citrate buffer (pH 6.0) and heated in a microwave oven to 95 °C for 20 min to facilitate antigen retrieval. Then, endogenous peroxidase activity was quenched, followed by blocking with normal goat serum. Then, ARIH2 and Ki67 antibodies were diluted with BSA according to the manufacturer’s instructions, and the antibodies were added to the paraffin sections and incubated overnight at 4 °C. Then, a horseradish peroxidase-linked secondary antibody was added and incubated with the sections, which was followed by the addition of DBA reagent. The results were observed under a microscope before counterstaining with hematoxylin [22, 23].

### Western blot

RIPA was used for cell lysis to extract protein from the cells, and then, proteins were denatured and separated. Proteins of different molecular weights were separated by SDS-polyacrylamide gel electrophoresis and electrotransferred to polyvinylidene difluoride membranes. The membrane is sealed with skimmed milk to detect the proteins, and BSA to detect the phosphorylated proteins, and the membrane sections were incubated sequentially with primary antibodies, and then, secondary antibodies. The membranes were exposed to ECL Reagent (Cell Signaling) and visualized by Western blot analysis detection system (Thermo Fisher, Shanghai, China).

### Quantitative and reverse transcription PCR

Total RNA was extracted from cells using TRIzol reagent. Then, 2 µg of RNA was reverse transcribed into cDNA. The normalized expression control was based on the glyceraldehyde-3-phosphate dehydrogenase value. Finally, mRNA expression was determined as the CT value. All quantitative primers are given in Table 2.

**Table 2:** RT-PCR primers.

ARIH2-forward(5′-3′)	TGTATGCAGTTTGTGCGAAAGG
ARIH2-reverse(5′-3′)	GCCATGCAAGAGACTCCCA
p21-forward(5′-3′)	CGATGGAACTTCGACTTTGTCA
p21-reverse(5′-3′)	GCACAAGGGTACAAGACAGTG

### Immunoprecipitation

First, 293FT cells were transfected with specific plasmid, and transfected cells were lysed in IP lysis buffer. Then proteins were extracted from the cells. Target protein was incubated overnight at 4 °C with a certain proportion of primary antibody. Then 50 µl protein A + G agarose beads was added to the protein-antibody mixed solution, and incubated at 4 °C for 4 h. Target proteins were connected to the beads after incubation, and the beads were washed with precooled PBS buffer to remove impurities. The protein samples were added with 40 µl 1×loading buffer and heat denatured. After these steps, the protein samples were separated by SDS-polyacrylamide gel electrophoresis and electrotransferred to polyvinylidene difluoride membranes. The membranes were incubated with primary antibodies overnight at 4 °C and then incubated with secondary antibodies for 2 h. Finally, the membranes were exposed and analyzed in a Chemiscope 6000 imaging system.

### Cell proliferation detection

For analysis of the proliferation of cells, 1 × 10^3^ cells were cultured in 96-well plates for 7 days. An MTT assay was performed to detect cell viability and growth curves. All experiments were independently performed three times.

### BrdU staining

For BrdU staining, 2 × 10^4^ cells were seeded on coverslips in 24-well plates. Then the cells were incubated with 10 μg/ml BrdU (Sigma) for 30 min and fixed in 4% paraformaldehyde for 15 min. After treatment with 1 mol/L HCl and blocking with 5% goat serum, the cells were incubated sequentially with primary antibodies against BrdU and Alexa Fluor^®^ 594 secondary antibody. DAPI (4′,6-diamidino-2-phenylindole) was used for nuclear staining. BrdU-positive cells were calculated from at least 10 randomly chosen microscopic fields by microscopy (Nikon 80i; Nikon Corporation, Tokyo, Japan).

### Plate cloning

For plate cloning, 1 × 10^3^ cells were seeded on coverslips in six-well plates. Then 2 weeks later, the cells were stained with crystal violet staining solution and scanned under a scanner. Subsequently, they were decolorized with absolute ethanol, and after sufficient shaking. The absorbance was measured at 560 nm, and the graph was plotted based on the absorbance.

### Soft agar assay

The solidified bottom layer was made with low-melting-point agar in a six-well plate. The top layer containing the mixture of 0.3% agar and GC cells was then laid on top of the bottom supporting agar layer. After 14–21 days of incubation, colonies in each well were photographed and counted.

### Flow cytometry

For cell cycle analysis, cells were harvested and fixed in 70% ethanol, stained with propidium iodide (PI) and analyzed by flow cytometry (BD Biosciences, San Jose, CA, USA). The data were analyzed with CellQuest software (BD Biosciences).

### Comet assay

A comet assay to detect DNA damage in cells was performed following the manufacturer’s instructions. Briefly, cells were harvested and adjusted to 1 × 10^5^ cells/ml, mixed with molten LM agarose at a ratio of 1:10 (V/V) and immediately pipetted into 50 µl of mixture onto a CometSlide. After gelling, lysis, electrophoresis and DNA precipitation, the slides were stained with Green-DNA Dye (#163795–75–3) from Sangon Biotech and analyzed by fluorescence microscopy.

### Immunofluorescence assay

An immunofluorescence assay was performed to detect the expression of ɣ-H2AX in GC cells. Briefly, cells were collected, fixed and opsonized. After blocking, the cells were incubated with an anti-ɣ-H2AX antibody (1:5000) at 4 °C overnight. The cells were then incubated with Alexa Fluor 594-labeled secondary antibody (1:2000). Hoechst 33342 (1:2000) was then used to stain the nuclei, and ɣ-H2AX-positive cells were captured under an Olympus FV1000 confocal fluorescence microscope (FluoView FV1000, Olympus, Japan).

### Xenograft assay

Animal experiments were approved by the Committee for Animal Protection and Utilization of Southwest University. All experiments were conducted in accordance with the Guidelines for Animal Health and Use (Ministry of Science and Technology, China, 2006). Four-week-old female NOD/SCID mice were purchased and housed in an SPF room that was maintained at a constant temperature and humidity. 1 × 10^5^ human GC cells (MKN45) stably transfected with shGFP, shARIH2#1, shARIH2#1/shGFP or shARIH2#1/shp21, were injected slowly into a single side of the armpit of each mouse. At the termination of the experiment, the tumors were removed, processed and analyzed. Randomization and single blinding were used for measurement. Finally, the tumors were collected and photographed for subsequent immunohistochemical staining as described previously.

### Ubiquitination assay

For the ubiquitination assay in vivo, 293FT cells were cotransfected with the indicated plasmids. At 48 h after transfection, the cells were treated with 50 μg/ml of the proteasome inhibitor MG132 for 6 h. The cells were then lysed in Cell Lysis Buffer (Sigma) for Western blot and IP following the same protocol used for co-IP.

### Turnover assay

The cells were transfected with the indicated plasmids. CHX was added to the media at final concentration of 60 μg/ml. The cells were harvested at the indicated time points after CHX treatment. The protein levels were analyzed by Western blot assay. The protein density was measured by a densitometer and the integrated optical density was measured.

### Patient data analysis and tumor samples

Patient data and gene expression datasets were obtained from the R2: microarray analysis and visualization platform (https://hgserver1.amc.nl/cgi-bin/r2/main.cgi). Kaplan–Meier analysis and generation of survival curves were performed by using GraphPad Prism (version 6.0, GraphPad Software, San Diego, CA, USA). All cutoff values for separating high and low expression groups were determined by the online R2 database algorithm. Tumor samples were purchased from Chaoying Biotechnology Co., Ltd. (Xian, China) and they were originally obtained from Tongxu County People’s Hospital of Henan Province. Written informed consent to participate was provided by all the patients.

### Statistical analysis

All experiments were carried out in triplicate, and statistical parameters, including the sample size and the significance analysis are specified in the figure legends. Two-tailed Student’s *t* test was performed to calculate significance with a 95% confidence level, following normal distribution with a different but similar s.d. The quantitative data are expressed as the mean ± s.d., and a value of *P* < 0.05 was considered statistically significant.

## Results

### High ARIH2 expression is correlated with poor GC patient prognosis

**Fig. 1:**
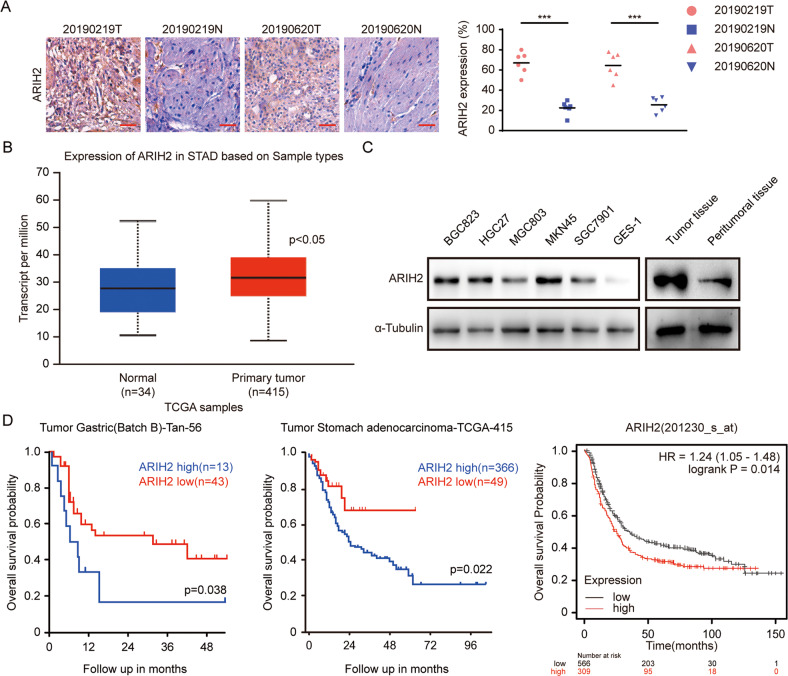
High ARIH2 expression is correlated with poor GC patient prognosis. **A** Immunohistochemical staining analysis showed the expression of ARIH2 and quantified the expression of ARIH2 in normal and GC tumor tissues. Scale bar = 50 μm. **B** Box plot of ARIH2 expression levels in the peritumoral tissues (normal) and GC tumors with log-rank test *P* values < 0.05. **C** Western blot assays were used to detect the protein expression of ARIH2 in GC tissues and peritumoral tissues (normal), as well as in the normal gastric cell line (GES-1) and GC cell lines (BGC823, HGC27, MGC803, MKN45, SGC7901). **D** Kaplan–Meier analysis of progression-free survival using data from three different GC databases. All data are expressed as the mean ± SD. Student’s *t* test was performed to analyze significance. *P < 0.05, **P < 0.01, ***P < 0.001.

To verify the relationship between the prognosis of GC patients and the expression of ARIH2, we performed immunohistochemical staining to detect the expression of ARIH2 in normal tissues and tumor tissue of GC patients. The results showed that the expression of ARIH2 in the tumor tissue of GC patients was higher than that in normal tissues (Fig. 1A). From The Cancer Genome Atlas (TCGA) database (https://portal.gdc.cancer.gov/), we found that ARIH2 was highly expressed in GC tissues, compared with normal tissues (Fig. 1B). Then, we detected the expression of ARIH2 in five GC cell lines, BGC823, HGC27, MGC803, MKN45, and SGC7901 cell lines and a normal gastric cell line GES-1, by Western blot assays. We found that ARIH2 expression in five GC cell lines was significantly higher than in the normal gastric cell line. We further detected the expression of ARIH2 in tumor tissues and peritumoral tissues, and found that expression of ARIH2 was significantly upregulated in the tumor tissues (Fig. 1C). In addition, high expression of ARIH2 indicated poor prognosis of GC patients in the three GC datasets (Fig. 1D). Taken together, the results validated that ARIH2 is highly expressed in GC tissues compared with normal tissues and may have a carcinogenic role in GC.

### Downregulation of ARIH2 expression inhibits cell proliferation and regulates the cell cycle progression of GC cells

**Fig. 2:**
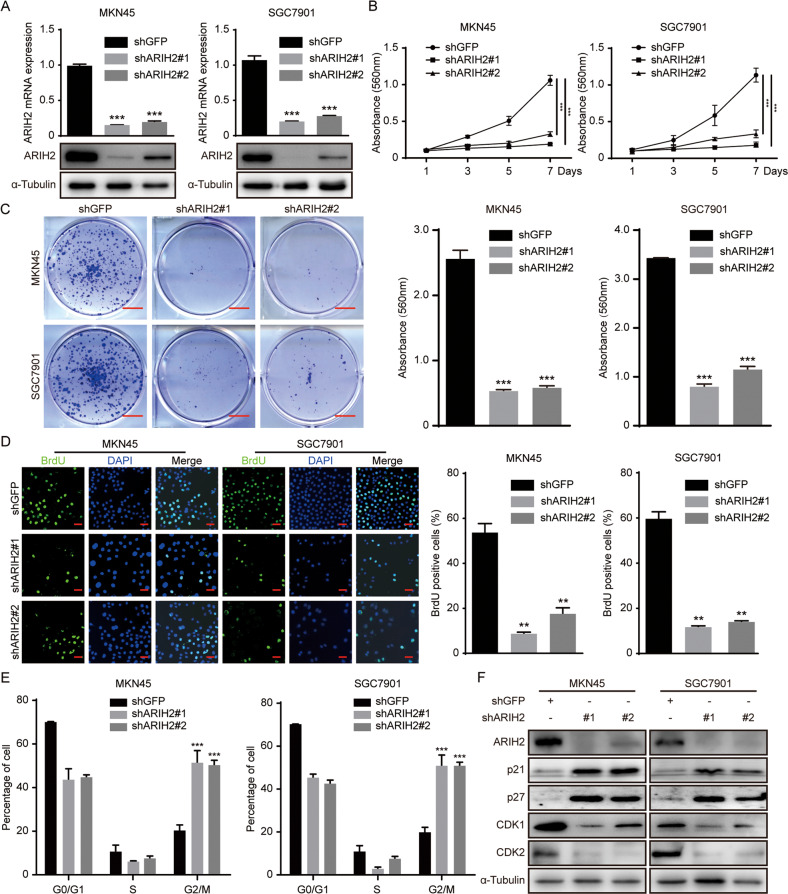
Downregulation of ARIH2 expression inhibits cell proliferation and regulates the cell cycle progression of GC cells. **A** Western blot and quantitative PCR assays were performed to characterize the expression of ARIH2 in the control and ARIH2-knockdown MKN45 and SGC7901 cells. **B** MTT assays were performed to test the proliferation of the control and ARIH2-knockdown MKN45 and SGC7901 cells. **C** Plate cloning assays were performed to examine the proliferation of the control and ARIH2-knockdown MKN45 and SGC7901 cells. **D** BrdU incorporation assays were performed to detect the amount of DNA synthesis in the control and ARIH2-knockdown MKN45 and SGC7901 cells. Data were analyzed using two-tailed Student’s *t* tests. Scale bar = 50 μm. **E**, **F** The cell cycle was analyzed in MKN45 and SGC7901 cells by flow cytometry, and cell cycle-related proteins were detected by Western blot assays. All data were expressed as the mean ± SD. Student’s *t* test was performed to analyze significance. *P < 0.05, **P < 0.01, ***P < 0.001.

To further explore the effect of ARIH2 on the proliferation of GC cells, we successfully knocked down ARIH2 expression in MKN45 and SGC7901 cells by treating them with lentiviruses carrying shRNA sequences (Fig. 2A). ARIH2-knockdown GC cells showed significant morphological changes, and the cell numbers were sharply decreased, as demonstrated by microscopy (Supplementary Fig. 1A). Next, we tested the effect of downregulated ARIH2 expression on the proliferation of MKN45 and SGC7901 cells by MTT assays, and the results revealed that ARIH2 knockdown obviously inhibited the proliferation of MKN45 and SGC7901 cells (Fig. 2B). Plate cloning assays were performed and showed that the cloning ability of GC cells was notably restrained following ARIH2 knockdown (Fig. 2C). The 5′-bromo-2-deoxyuridine (BrdU) assay results consistently showed that the BrdU-positive rates in the shARIH2 groups were much lower than those observed in the corresponding control groups (Fig. 2D). Then, we examined the cell cycle distribution of the ARIH2-knockdown and control cells by flow cytometry and observed that ARIH2 knockdown induced cell cycle arrest at G2/M phase (Fig. 2E). To further verify the results above, we measured and analyzed G2/M phase-related proteins by Western blot assays. The results showed that the expression of p21 and p27 was increased and the expression of CDK1 and CDK2 was decreased after ARIH2 knockdown (Fig. 2F). Moreover, elevated p21 mRNA expression was also detected in the ARIH2-knockdown GC cells by qRT-PCR (Supplementary Fig. 1B). In addition, we overexpressed ARIH2 in MKN45 and SGC7901 cells. The results showed ARIH2 overexpression could significantly decrease p21 and p27 expression and increase CDK1 and CDK2 expression. MTT and BrdU assays were subsequently performed and indicated that ARIH2 overexpression could significantly facilitate the proliferation of MKN45 and SGC7901 cells (Supplementary Fig. 2A–C). Thus, ARIH2 is essential for the proliferation of GC cells.

### ARIH2 is responsible for colony formation of GC cells in vitro and for tumor formation of GC cells in vivo

**Fig. 3:**
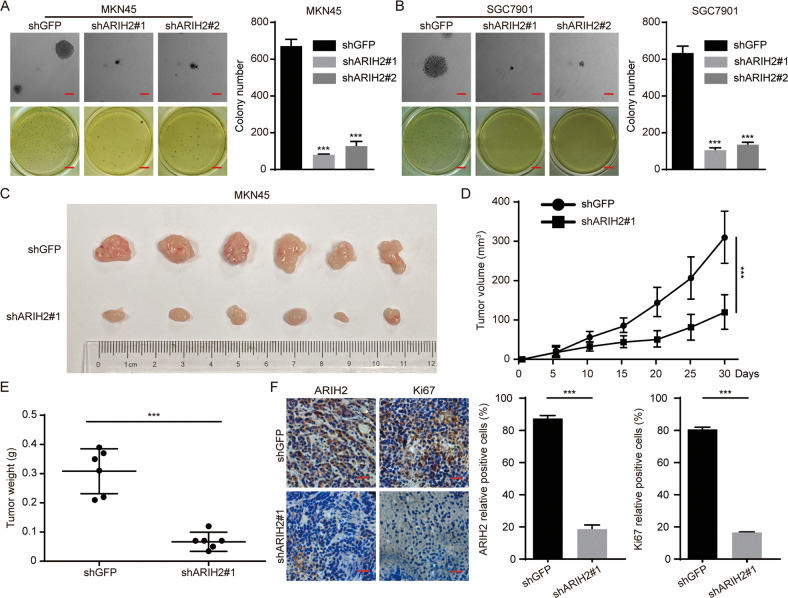
ARIH2 is responsible for colony formation of GC cells in vitro and for tumor formation of GC cells in vivo. **A**, **B** Soft agar assays of the control and ARIH2-knockdown MKN45 and SGC7901 cells were performed to detect the colony formation ability. Quantification of the number of cell clones. **C**–**E** Photographs, growth monitoring and weights of the indicated xenograft tumors. Data were analyzed using two-tailed Student’s *t* tests. **F** IHC analysis of ARIH2 and Ki67 expression was carried out in the indicated xenograft tumors. Scale bar = 50 μm. Quantification of ARIH2 and Ki67 expression. All data are expressed as the mean ± SD. Student’s *t* test was performed to analyze significance. *P < 0.05, **P < 0.01, ***P < 0.001.

Subsequently, we examined the clonogenic abilities of GC cells after ARIH2 knockdown by soft agar assays in vitro. The results showed that clones of the ARIH2-knockdown GC cells were significantly fewer and smaller than those of the control GC cells (Fig. 3A, B). Next, subcutaneous xenograft experiments indicated that the growth rate and the volume and weight of the tumors formed by the ARIH2-knockdown MKN45 cells were significantly decreased compared with those of the control MKN45 cells (Fig. 3C, D, E). To determine whether ARIH2 enhances the tumor progression by promoting the proliferation of GC cells, we performed immunohistochemical staining and the results showed that the expression of ARIH2 and Ki67 in the ARIH2-knockdown tumors was significantly decreased (Fig. 3F). Taken together, these data indicated that ARIH2 is indispensable for colony formation and tumorigenesis of GC cells.

### ARIH2 interacts with p21 and regulates the expression of p21 via ubiquitination in an E3 ligase activity dependent manner

**Fig. 4:**
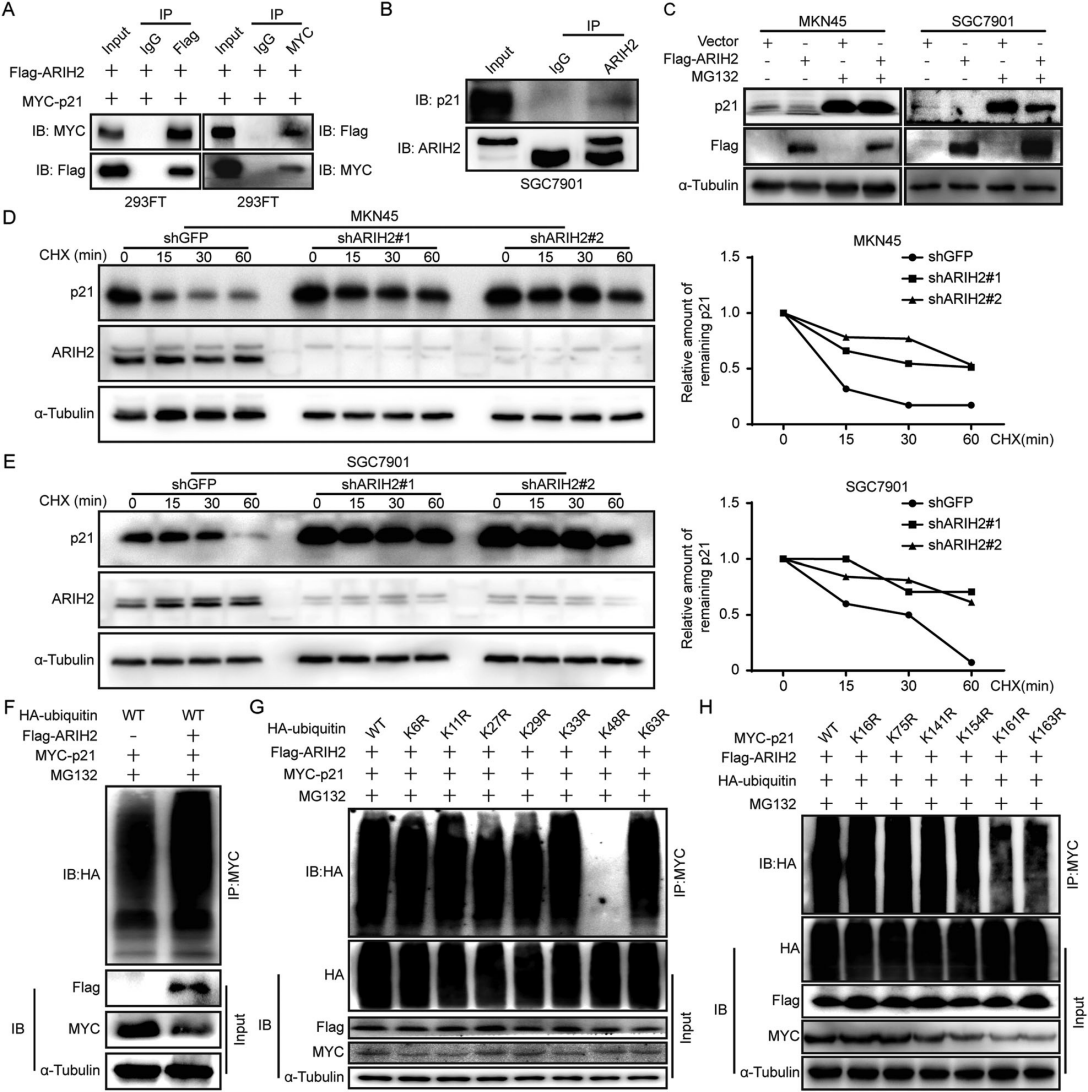
ARIH2 interacts with p21 and regulates the expression of p21 via ubiquitination in an E3 ligase activity dependent manner. **A** Flag-ARIH2 and MYC-p21 plasmids were cotransfected into 293FT cells, and the total cell lysates were immunoprecipitated with anti-Flag and anti-MYC antibodies, respectively. Then, anti-MYC and anti-Flag antibodies were used to detect the immunoprecipitates, and anti-Flag and anti-MYC antibodies were used to test the success of the experiment. **B** SGC7901 cell lysates were subjected to immunoprecipitation with control IgG and ARIH2 antibodies. The immunoprecipitate was then probed with p21 antibody, and the ARIH2 antibody was used to detect the success of the experiment. **C** Cell lysates were prepared from the control and ARIH2-overexpressing GC cells that had been treated with MG132 for 6 h. Equal amounts of cell lysates were immunoblotted with the indicated antibodies. **D**, **E** The control and ARIH2-knockdown GC cells were treated with CHX (100 μg/ml) for the indicated times. Cell lysates were immunoblotted with the indicated antibodies. Quantification of the turnover of p21 in the control and ARIH2-knockdown GC cells. **F** Flag-ARIH2, MYC-p21 and HA-ubiquitin plasmids were transiently transfected into 293FT cells for 2 d, and the transfected 293FT cells were treated with MG132 for 7 h before proteins were harvested. The ubiquitinated p21 proteins were pulled down with an anti-MYC antibody and immunoblotted with an anti-HA antibody. **G** HA-ubiquitin mutants in which only one lysine residue was mutated to an arginine residue, as shown, were used, and co-IP was performed. **H** MYC-p21 mutants in which only one lysine residue was mutated to an arginine residue, as shown, were used, and co-IP was performed.

Our experiments above showed that p21 expression were significantly increased after knocking down ARIH2. Given that p21 can be rapidly degraded via ubiquitination and ARIH2 is an E3 ligase, we investigated whether ARIH2 interacts with p21 and whether ARIH2 regulates p21 ubiquitination. First, we explored the relationship between ARIH2 and p21 in 293FT and SGC7901 cell lines by coimmunoprecipitation (co-IP). The results showed that p21 was detected in Flag-ARIH2 immunoprecipitates and that ARIH2 was also found in MYC-p21 immunoprecipitates (Fig. 4A). Moreover, the co-IP results demonstrated that endogenous ARIH2 and p21 also coprecipitated reciprocally (Fig. 4B). These results indicated that a protein interaction is present between ARIH2 and p21. Next, the proteasome inhibitor MG132 was added to the ARIH2-overexpressing MKN45 and SGC7901 cells. Western blot assays showed that p21 expression was significantly decreased after ARIH2 overexpression while increased after MG132 addition, indicating that ARIH2 regulated p21 at the post-transcriptional level (Fig. 4C). Then, the de novo protein synthesis inhibitor cycloheximide (CHX) was added to the control and ARIH2-knockdown GC cells to detect the turnover of p21. The results showed that the turnover of p21 was prolonged after downregulation of ARIH2 expression, which proved that ARIH2 could regulate the stability of p21 (Fig. 4D, E). To further probe the potential mechanism of the regulation of p21 stability by ARIH2, we examined the effect of ARIH2 on p21 ubiquitination by co-IP. MYC-tagged p21, HA-tagged ubiquitin and Flag-tagged ARIH2 were cotransfected into 293FT cells. The results showed that the level of p21 ubiquitination was increased after ARIH2 overexpression (Fig. 4F). In addition, an in vitro ubiquitination assay was performed, and the results showed that ARIH2 directly ubiquitinates p21 (Supplementary Fig. 3A). These data demonstrated that ARIH2 directly regulates p21 via ubiquitination and that ARIH2 is an E3 ligase for p21.

To identify the key amino acid residues of ubiquitin in ARIH2-mediated p21 ubiquitination, we cotransfected MYC-tagged p21, Flag-tagged ARIH2 and HA-tagged wild-type or mutant ubiquitin, in which one lysine was mutated into arginine (K6R, K11R, K27R, K29R, K33R, K48R, K63R) to inactivate the site, into 293FT cells. The results showed that the level of p21 ubiquitination was significantly decreased by K48R but not by K6R, K11R, K27R, K29R, K33R or K63R (Fig. 4G). Next, to identify the key amino acid residues of p21 in ARIH2-mediated p21 ubiquitination, we constructed several MYC-tagged p21 mutants with single point mutations (K16R, K75R, K141R, K154R, K161R, K163R), and co-IP was also performed. The results showed that the level of p21 ubiquitination was significantly decreased by K161R but not by K16R, K75R, K141R, K154R, or K163R (Fig. 4H). These results indicated that ARIH2 regulates p21 ubiquitination and that the K48 residue of ubiquitin and the K161 residue of p21 play key roles in ARIH2-regulated p21 ubiquitination.

### Downregulation of p21 expression in ARIH2-knockdown GC cells partially restores the inhibition of proliferation induced by ARIH2 knockdown

**Fig. 5:**
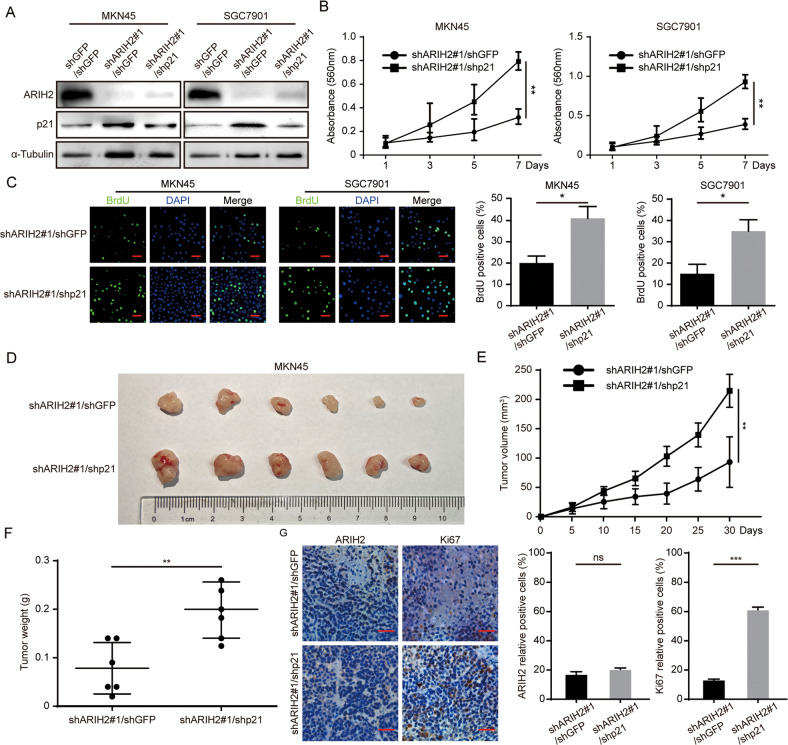
Downregulation of p21 expression in ARIH2-knockdown GC cells partially restores the inhibition of proliferation induced by ARIH2 knockdown. **A** Western blot analysis of protein after knocking down p21 in the ARIH2 knockdown GC cells was performed. **B** MTT assays were performed to detect the proliferation of GC cells. **C** BrdU incorporation assays were performed to detect the amount of DNA synthesis. Data were analyzed using two-tailed Student’s *t* tests. Scale bar = 50 μm. **D**–**F** Photographs, growth monitoring and weights of the indicated xenograft tumors. Data were analyzed using two-tailed Student’s *t* tests. **G** IHC analysis of ARIH2 and Ki67 expression was carried out in the indicated xenograft tumors. Quantification of ARIH2 and Ki67 expression. Scale bar = 50 μm. All data are expressed as the mean ± SD. Student’s *t* test was performed to analyze significance. *P < 0.05, **P < 0.01, ***P < 0.001.

We have shown that ARIH2 directly interacts with p21, and that ARIH2 can regulate the expression of p21 via ubiquitination. We confirmed the key amino acid residues by ubiquitination-related assays. Next, we knocked down p21 in the ARIH2-knockdown GC cells and examined the effect on the proliferation of GC cells. Western blot assays were performed to detect ARIH2 and p21 expression after downregulation of p21 expression in the ARIH2-knockdown GC cells (Fig. 5A). Then, we tested the proliferation of GC cells by MTT and BrdU assays and found that downregulated p21 expression after ARIH2 knockdown could significantly restore the inhibition of proliferation caused by ARIH2 knockdown (Fig. 5B, C). We also performed subcutaneous xenograft experiments, and the results indicated that the growth rate and the volume and weight of the tumors were significantly restored (Fig. 5D, E, F). Next, immunohistochemical staining was performed on tumor samples from mice with subcutaneous tumors and showed that the rate of Ki67-positive cells was significantly rescued after downregulation of p21 expression in the ARIH2-knockdown GC cells (Fig. 5G). To further confirm the regulatory effect of ARIH2 on p21 and examine the key role played by K161 of p21, we transfected wild-type or K161R mutant MYC-p21 into MKN45 and SGC7901 cells after ARIH2 overexpressing. Plate cloning and MTT assays showed that the enhanced proliferation caused by ARIH2 overexpression was inhibited after wild-type MYC-p21 transfection, while the K161R mutant of MYC-p21 lost this effect (Supplementary Fig. 2D, E). Moreover, we detected E3 ligase SKP2, RNF126 and UHRF2 expression after ARIH2 knockdown by Western blot assays. Studies have shown that SKP2, RNF126 and UHRF2, interacting with p21 and causing the degradation of p21, are E3 ligases for p21. The results showed that ARIH2 knockdown in GC cells also significantly reduced SKP2, RNF126 and UHRF2 expression, which indicates that ARIH2 may not be the only factor regulating p21 expression (Supplementary Fig. 3B). These data showed that p21 is the substrate protein to which ARIH2 binds and that ARIH2 regulates the proliferation of GC cells by regulating p21 expression.

### Downregulation of ARIH2 expression induces DNA damage and apoptosis in GC cells

**Fig. 6:**
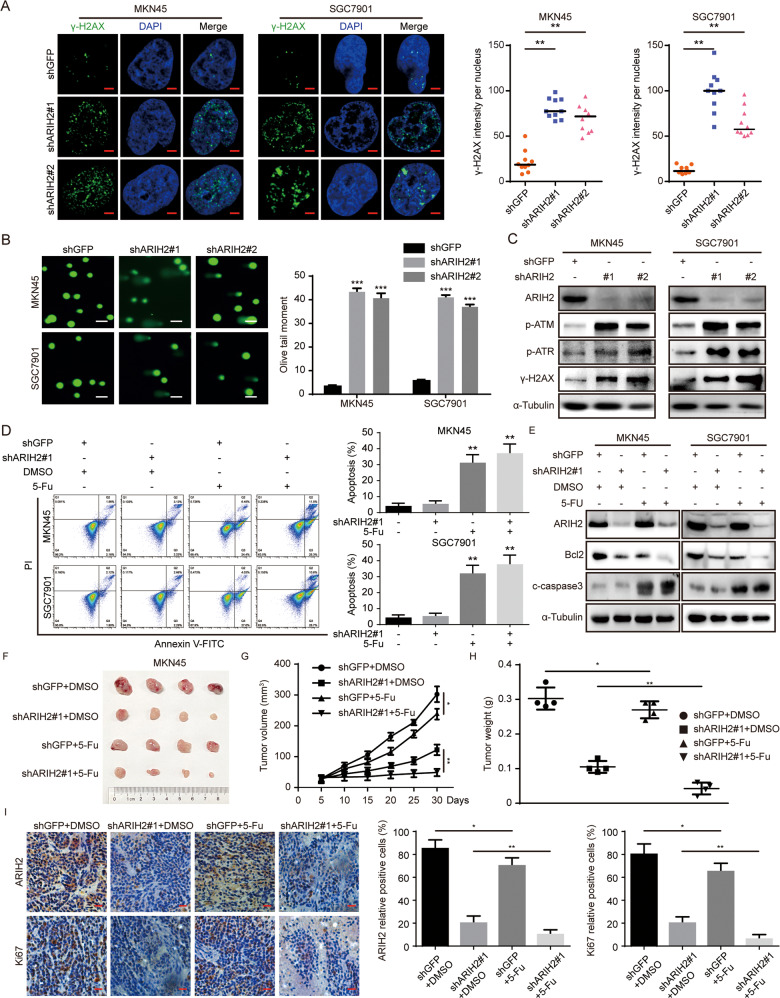
Downregulation of ARIH2 expression induces DNA damage and apoptosis in GC cells. **A** Immunofluorescence staining with a ɣ-H2AX antibody was performed to confirm the induction of DNA damage after ARIH2 knockdown. Representative ɣ-H2AX-positive cells are shown. Quantification of ɣ-H2AX expression. **B** Tailed DNA in single GC cells with ARIH2 knockdown was detected. Quantification of tailed DNA in single cells. **C** DNA damage-related proteins were analyzed by Western blot assays. **D** Flow cytometric analysis of apoptosis of the GC cells with ARIH2 knockdown were performed. Quantification of apoptosis of GC cells. **E** Apoptosis-related proteins were detected by Western blot assays. **F**–**H** Photographs, growth monitoring and weights of the indicated xenograft tumors. Data were analyzed using two-tailed Student’s *t* tests. **I** IHC analysis of ARIH2 and Ki67 expression was carried out in the indicated xenograft tumors. Scale bar = 50 μm. Quantification of ARIH2 and Ki67 expression. All data are expressed as the mean ± SD. Student’s *t* test was performed to analyze significance. *P < 0.05, **P < 0.01, ***P < 0.001.

According to the results above, ARIH2 could promote the proliferation of GC cells in vivo and in vitro, and ARIH2 regulates p21 expression via ubiquitination. Studies have demonstrated that ARIH2 induces apoptosis and clonogenic inhibition in myeloid cells with RING-dependent E3 ligase activity and that ARIH2 inhibits cell and clone growth by inducing apoptosis in several cancer cell lines. Therefore, we speculated that ARIH2 may also play a key role in DNA damage and subsequent apoptosis of GC cells. To test this hypothesis, we performed an immunofluorescence assay. The results showed that ARIH2 knockdown caused elevation of the ɣ-H2AX signal in MKN45 and SGC7901 cells (Fig. 6A). Using the neutral comet assay, we subsequently evaluated the overall effect of ARIH2 on DNA damage at the individual cell level. We observed that the length of the comet tails of the ARIH2-knockdown GC cells was increased significantly compared to that of the control group, indicating that ARIH2 knockdown significantly induces DNA damage (Fig. 6B). Western blot assays were further used to verify the role of ARIH2 in DNA damage, showing that the expression of the DNA damage-related proteins phosphorylated(p)-ATM, p-ATR and ɣ-H2AX was significantly increased after ARIH2 knockdown (Fig. 6C). To confirm that ARIH2 is involved in the regulation of chemotherapeutic sensitivity, we used the chemotherapy agent 5-fluorouracil to treat GC cells. Flow cytometry was performed to detect the apoptosis of GC cells. The results showed that the degree of apoptosis was increased after the treatment of 5-fluorouracil, while the ARIH2-knockdown GC cells treated with 5-fluorouracil showed a higher degree of apoptosis (Fig. 6D). To further verify the results above, we performed Western blot assays. The level of the apoptosis-related protein Bcl2 was significantly reduced, that of the apoptosis marker cleaved caspase3 was increased, and treatment with 5-fluorouracil increased the significance of the changes in Bcl2 and cleaved caspase3 (Fig. 6E). In addition, subcutaneous xenograft experiments were performed, showing that the growth rate and the volume and weight of the tumors with ARIH2 knockdown and 5-fluorouracil treatment were significantly inhibited (Fig. 6F, G, H). Immunohistochemical staining was also performed and showed that the rate of Ki67-positive cells was significantly inhibited in the GC tumor formed by ARIH2-knockdown GC cells treated by 5-fluorouracil (Fig. 6I). Taken together, the results showed that ARIH2 is involved in DNA damage and subsequent apoptosis and that the treatment of 5-fluorouracil increased the degree of apoptosis in the ARIH2-knockdown GC cells.

## Discussion

Gastric cancer (GC) is one of the most common malignancies worldwide, and is characterized by recurring attacks and high levels of invasion into the surrounding normal tissue and vascularization [24]. Despite its worldwide decline in incidence over the past century, GC remains a major cause of death globally. Therefore, there is an urgent need to probe the deep molecular mechanism of GC. ARIH2 is a member of the Ariadne subfamily in the E3 ubiquitin ligase family, encoding a two RING fingers and double RING dinger linked 1 (TRIAD1) protein with E3 ubiquitin ligase activity [7, 25]. Prior research showed that ARIH2 promotes the ubiquitination of nucleotide-binding oligomerization domain-like receptor family pyrin domain containing 3 (NLRP3) and suppresses NLRP3 inflammasome activation in macrophages [6]. ARIH2 also inhibited the development of acute myeloid leukemia induced by the MLL1 fusion protein [5]. The interaction relationships between RING1 and UbcH7, RING2 and Ubc13 have been reported in the literature, indicating that both RING domains of ARIH2 play major roles in myeloid cell proliferation inhibition [26]. Moreover, increased expression of ARIH2 was correlated with neuronal apoptosis after intracerebral hemorrhage in adult rats, indicating that ARIH2 is an interventional target of secondary damage following ICH [27]. However, the biological functions of ARIH2 in GC cells remain unclear.

In this paper, we discovered that ARIH2 is highly expressed in GC cell lines and negatively correlates with the prognosis of GC patients. For measurement of the proliferation of the control and ARIH2-knockdown GC cells, Western blot, MTT, BrdU, plate cloning, soft agar and tumor xenograft analysis were successively performed. The results demonstrated that the proliferation of GC cells was obviously suppressed after ARIH2 knockdown. Next, the protein interaction between ARIH2 and p21 was confirmed by co-IP, and K48 of ubiquitin and K161 of p21 were the key amino acid residues in p21 ubiquitination by ARIH2. In addition, we found that the level of DNA damage was obviously increased after ARIH2 knockdown and that ARIH2-knockdown GC cells showed a higher degree of apoptosis after the combined treatment with 5-fluorouracil, indicating that ARIH2 is involved in DNA damage and that ARIH2 regulates chemosensitivity of GC cells. Thus, ARIH2 is indispensable for the proliferation of GC cells, and ARIH2 regulates the proliferation of GC cells by regulating p21 expression via ubiquitination.

Although ARIH2 is highly expressed in GC, the biological molecular mechanism of ARIH2 in the development and maintenance of GC remains elusive, largely due to the sophisticated pathogenesis and unclear environmental factors [28]. ARIH2 is an RBR E3 ligase, notable for its peculiar RBR domain. RBR E3 ligases are a class of E3 ligases, characterized by the unique RING-HECT hybrid, combining properties of RING and HECT-type E3s and undergoing multilevel regulation through autoinhibition, post-translational modifications, multimerization and interaction with binding partners [29, 30]. RBR E3 ligases perform different functions in a variety of human cancers. Previous studies have reported that RNF14, RNF31, RNF144B, RNF216 and ARIH1 mainly play carcinogenic roles, while ARIH2 and PARK2 generally play anticancer roles in malignant tumors [31, 32]. These molecules have been shown to be associated with important cellular events including protein stability and degradation, subcellular tethering, transcription and translation, and cellular signaling. Here we discovered that ARIH2 promotes the proliferation of GC cells and may play a carcinogenic role. Our studies showed that the protein expression of p21 was increased after downregulation of ARIH2 expression, and that ARIH2 regulated p21 via ubiquitination. However, we detected the expression of E3 ligases SKP2, RNF126 and UHRF2, which regulate p21 expression via ubiquitination, and found that ARIH2 knockdown changed the expressions of SKP2, RNF126 and UHRF2, indicating that ARIH2 may cooperate with other factors to regulate the expression of p21, while the mechanism needs to be further explored. Moreover, an in vitro ubiquitination assay showed that ARIH2 directly regulates p21, proving that ARIH2 is an E3 ligase of p21. K48-linked ubiquitination regulates protein degradation, whereas K63-linked ubiquitination regulates protein trafficking and signaling [33]. In this paper, we showed that K48 of ubiquitin was required for ARIH2-mediated p21 ubiquitination, which is consistent with previous studies. Prior evidence showed that RING2 contributes to the E3 ubiquitin ligase activity of the RING-IBR-RING motif, and a conserved cysteine in RING2 forms a transient thioester bond with ubiquitin, facilitating subsequent ubiquitin ligation to a lysine [25, 34]. It has been reported that C300, which is located in the RING2 domain encoded by the 297th to 326th amino acid residues in ARIH2, plays a key role in the ubiquitination of NLRP3 [6]. These results above indicated that the RING2 domain is indispensable in ARIH2-mediated ubiquitination. The key domains of ARIH2-mediated p21 ubiquitination will also be further verified in our experiments. In addition, it has been reported that ARIH2 can induce apoptosis in several cancer cell lines including MCF7, A549 and U2OS, via its RING ligase activity [27, 35]. Our research also indicated that ARIH2 is involved in the regulation of DNA damage and chemosensitivity of GC cells, and the apoptosis of ARIH2-knockdown GC cells was increased after combined treatment with 5-fluorouracil. However, the underlying mechanism needs to be further probed.

In general, our study showed that ARIH2 facilitated the proliferation of GC cells. The directly protein interaction between p21 and ARIH2 was first confirmed, and key amino acid residues of p21 and ubiquitin in ARIH2-mediated p21 ubiquitination were identified. Our research provides relevant insights for the identification of GC pathogenesis and the determination of GC treatment targets.

## Supplementary information

Supplemental figure

Reproducibility checklist

Detailed author contributions

Original Data File

Original Data File

Original Data File

Original Data File

Original Data File

Original Data File

Original Data File

## Data Availability

All of the data and material in this paper are available when requested.
