# Supplementary material for: ARIH2 regulates the proliferation, DNA damage and chemosensitivity of gastric cancer cells by reducing the stability of p21 via ubiquitination
Source: Cell Death Dis. 2022 Jun 22;13(6):564. doi: 10.1038/s41419-022-04965-9 (PMC9218151; doi:10.1038/s41419-022-04965-9)
Supplement: Supplementary file 1 — Supplemental figure [file 41419_2022_4965_MOESM1_ESM.docx]

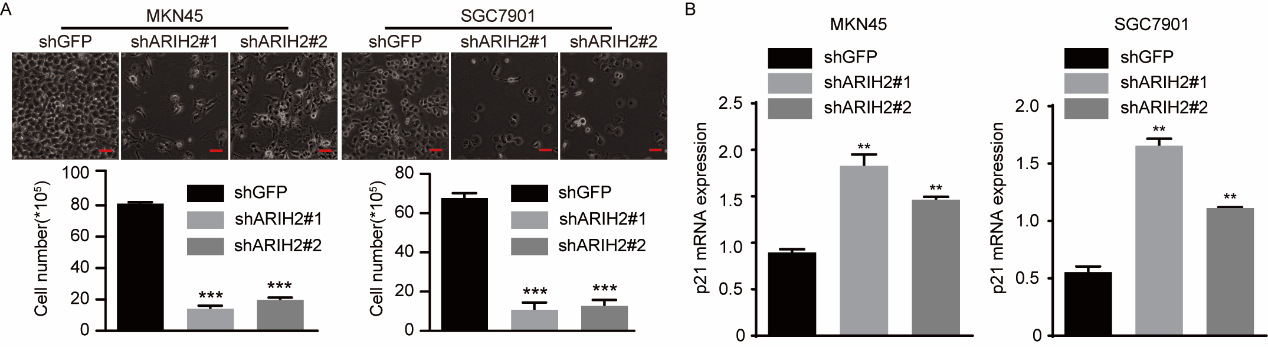


**Supplementary Fig. 1 A** Microscopic photograph of the control and ARIH2-knockdown GC cells. Scale bar=50 μm. **B** p21 mRNA expression of ARIH2-knockdown GC cells was quantified by qRT-PCR. All data were expressed as the mean ± SD. Student’s t test was performed to analyzed significance. *P< 0.05, **P< 0.01, ***P< 0.001.


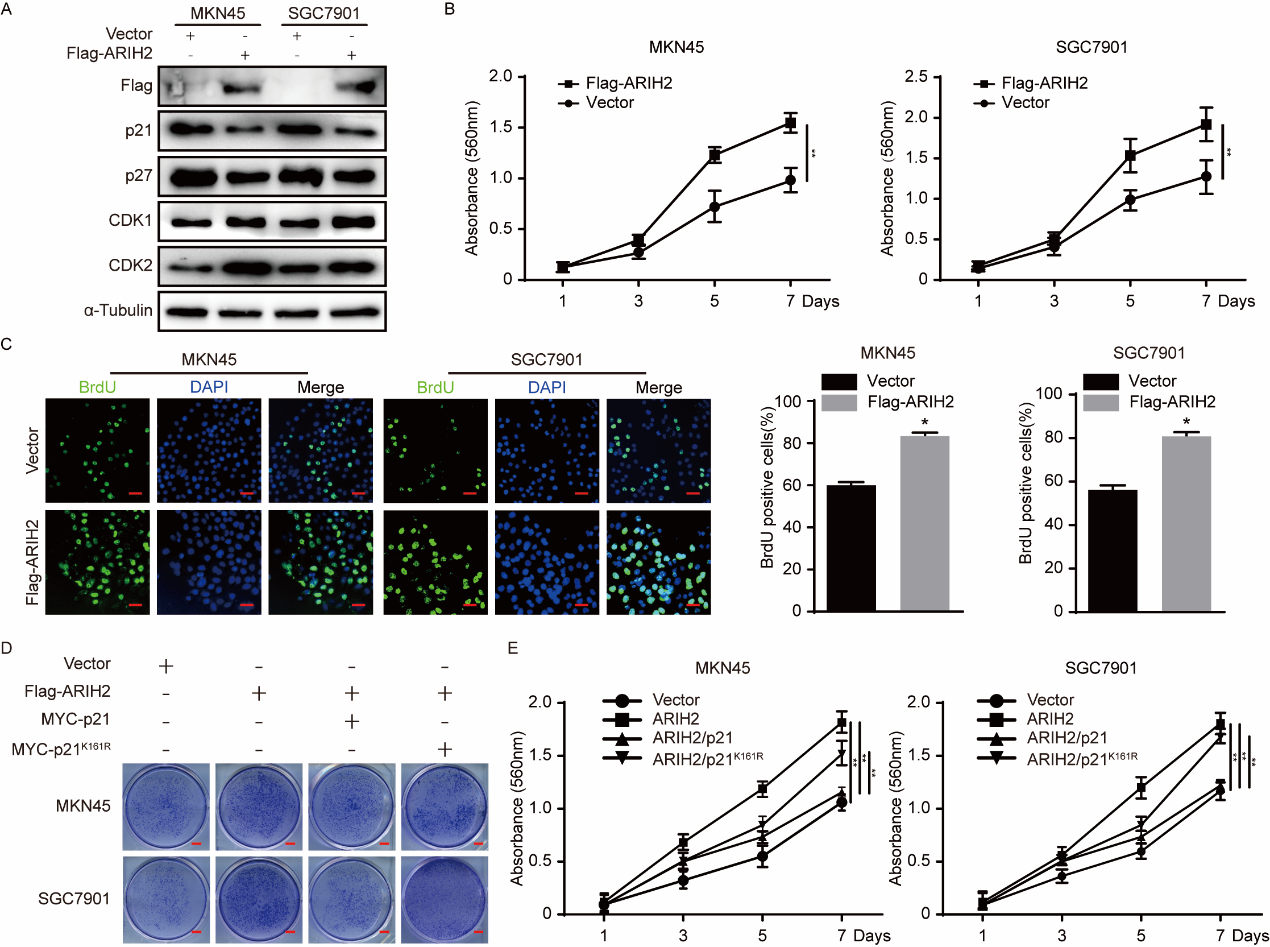


**Supplementary Fig. 2 A** Western blot assays were performed to detect the expression of G2/M phase-related proteins in ARIH2-overexpressing GC cells. **B, C** MTT and BrdU assays were performed to test the proliferation of ARIH2-overexpressing GC cells. Scale bar=50 μm. **D, E** Plate cloning and MTT assays were performed to detect the proliferation after the wild-type or K161R MYC-p21 plasmids were stably transfected into the ARIH2-overexpressing GC cells. All data were expressed as the mean ± SD. Student’s t test was performed to analyzed significance. *P< 0.05, **P< 0.01, ***P< 0.001.


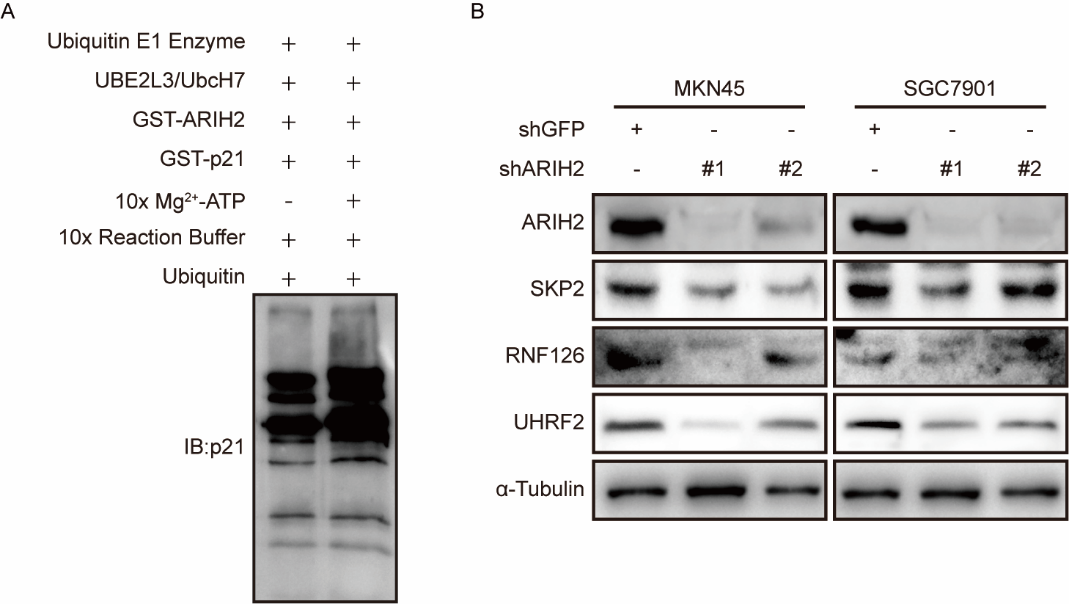


**Supplementary Fig. 3 A** An in vitro ubiquitination assay was performed to confirm the directly interaction between ARIH2 and p21. **B** Western blot assays were performed to detect SKP2, RNF126 and UHRF2 expression in the control and ARIH2-knockdown GC cells.
