# Supplementary material for: ARIH2 regulates the proliferation, DNA damage and chemosensitivity of gastric cancer cells by reducing the stability of p21 via ubiquitination
Source: Cell Death Dis. 2022 Jun 22;13(6):564. doi: 10.1038/s41419-022-04965-9 (PMC9218151; doi:10.1038/s41419-022-04965-9)
Supplement: Supplementary file 3 — Detailed author contributions [file 41419_2022_4965_MOESM3_ESM.pdf]

# DECLARATION OF CONTRIBUTIONS TO ARTICLE

**ADMC**

| | | |
|---|---|---|
| Manuscript Number: | Journal Name: | |
| CDDIS-21-3722R | *Cell Death & Disease* | (the 'Journal') |
| Proposed Title of the Contribution: | | |
| ARIH2 Regulates the Proliferation, DNA Damage and Chemosensitivity of Gastric Cancer Cells by Reducing the Stability of p21 via Ubiquitination | | (the 'Contribution) |
| Author(s): | | |
| Shengjun Geng, Wen Peng, Xue Wang, Xiaosong Hu, Hanghua Liang, Jianbing Hou, Feng Wang, Gaichao Zhao, Muhan Lü* and Hongjuan Cui* | | (the 'Authors') |

For all *CDDis* articles, each person named as an author in the published version must be able to show he or she has contributed substantially to the article.

Authorship credit should be based on 1) substantial contributions to conception and design, acquisition of data, or analysis and interpretation of data; 2) drafting the article or revising it critically for important intellectual content; and 3) final approval of the version to be published. Authors should meet conditions 1, 2 and 3.

Any person who cannot be shown to have made a substantial contribution to the article cannot be listed as an author in the final version. The name of any person who is deemed to have made a minor contribution can, however, appear in the Acknowledgments section of the article.

Please complete the table below to indicate the contributions of all named authors to the manuscript.

| Author Full Name: | Specification of Contribution to the Manuscript: |
|---|---|
| Shengjun Geng | Shengjun Geng designed experiments, obtained and analyzed data, and wrote the manuscript. |
| Wen Peng | Wen Peng helped design experiments, obtained and analyzed data, and revise the manuscript. |
| Xue Wang | Xue Wang helped obtain, analyze some of the data, and revise the manuscript. |
| Xiaosong Hu | Xiaosong Hu helped obtain, analyze some of the data, and revise the manuscript. |
| Hanghua Liang | Hanghua Liang helped obtain, analyze some of the data, and revise the manuscript. |
| Jianbing Hou | Jianbing Hou helped obtain, analyze some of the data, and revise the manuscript. |
| Feng Wang | Feng Wang designed experiments and revised the manuscript. |
| Gaichao Zhao | Gaichao Zhao designed experiments and revised the manuscript. |
| Muhan Lü | Muhan Lü read and revised this manuscript. |
| Hongjuan Cui | Hongjuan Cui read and revised this manuscript. |
| | |
| | |
| | |

Please complete the table below to indicate the contributions of all named authors to the figures.

Figure 1:

In Figure 1, SG and WP analyzed the data and prepared panel B and D; XW, XH and HL generated immunehistochemistry data, labelled the image and prepared panel A; XH and HL generated the WB data and prepared panel C.

Figure 2:

In Figure 2, SG and WP generated the WB and qRT-PCR data and prepared panel A and F;.JH, FW and GZ generated MTT, BrdU, plate cloning assays and flow cytometry data and prepared panel B-E.

Figure 3:

In Figure 3, WP generated the colony formation data and prepared panel A and B; SG, XW, XH and FW generated the xenograft assays and prepared the panel C-F.

Figure 4:

In Figure 4, SG, JH, FW and WP generated the WB data and prepared panel A-H.

Figure 5:

In Figure 5, JH generated the WB data and prepared panel A; SG, WP and XH generated MTT and BrdU data and prepared panel B and C; HL and FW generated xenograft assays and prepared the panel D-G.

Figure 6:

In Figure 6, SG generated the WB data and prepared panel C and E; WP, HL and FW generated IF and Comet data and prepared panel A and B; XH and GZ generated xenograft assays and flow cytometry data, and prepared the panel D and F-I.

| Signed for and on behalf of the Author(s): | Print Name: | Date: |
| --- | --- | --- |
| Hongjuan Cui | HONGJUAN CUI | Jan 24, 2022 |
| Shengjun. Geng | SHENGJUN GENG | 2022.1.24 |
| Wen Peng. | WEN PENG | 2022.1.24 |
| Xue Wang | XUE WANG | 2022.1.24 |

| | | |
|---|---|---|
| Xiaosong Hu | XIAOSONG HU | 2022.1.24 |
| Hanghua Liang | HANGHUA LIANG | 2022.1.24 |
| Jianbing Hou | JIANBING HOU | 2022.1.24 |
| Feng Wang | FENG WANG | 2022.1.24 |
| Gaichao Zhao | GAICHAO ZHAO | 2022.1.24 |
| Muhan Lü | MUHAN Lü | 2022.1.24 |
