# Supplementary figures and images for: ARIH2 regulates the proliferation, DNA damage and chemosensitivity of gastric cancer cells by reducing the stability of p21 via ubiquitination
Source: Cell Death Dis. 2022 Jun 22;13(6):564. doi: 10.1038/s41419-022-04965-9 (PMC9218151; doi:10.1038/s41419-022-04965-9)

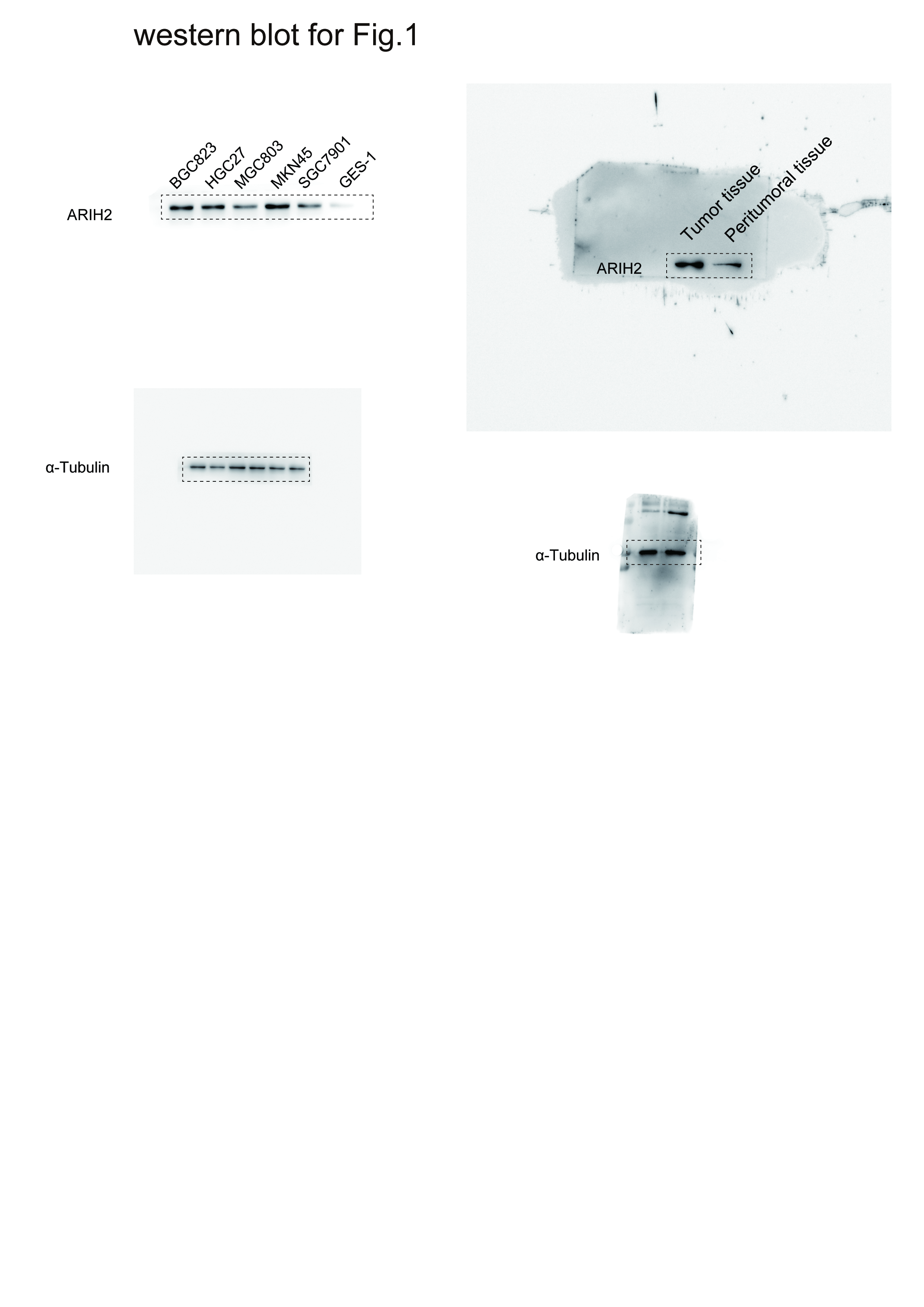

Supplement: Supplementary file 4 — Original Data File [file 41419_2022_4965_MOESM4_ESM.tif]

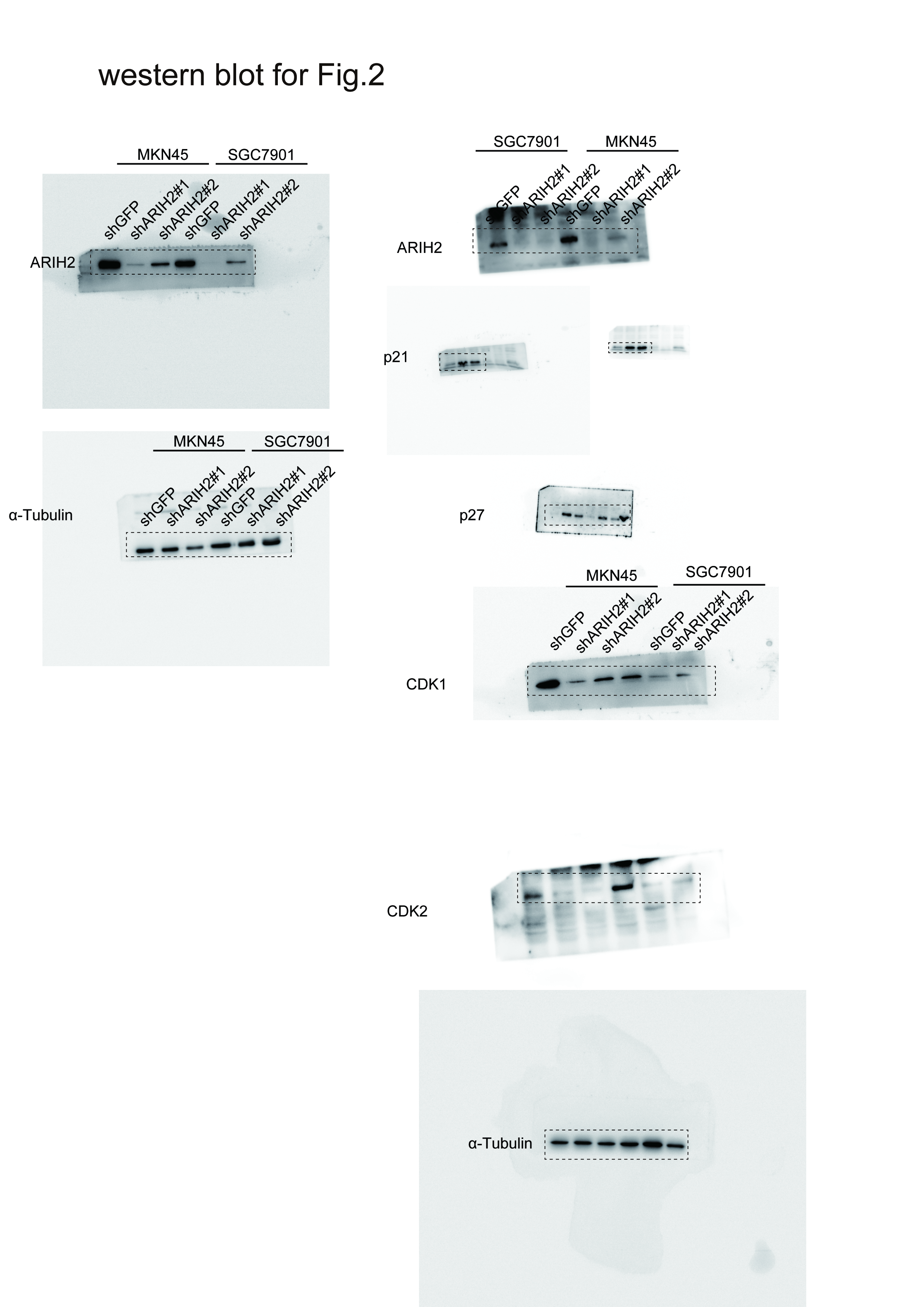

Supplement: Supplementary file 5 — Original Data File [file 41419_2022_4965_MOESM5_ESM.tif]

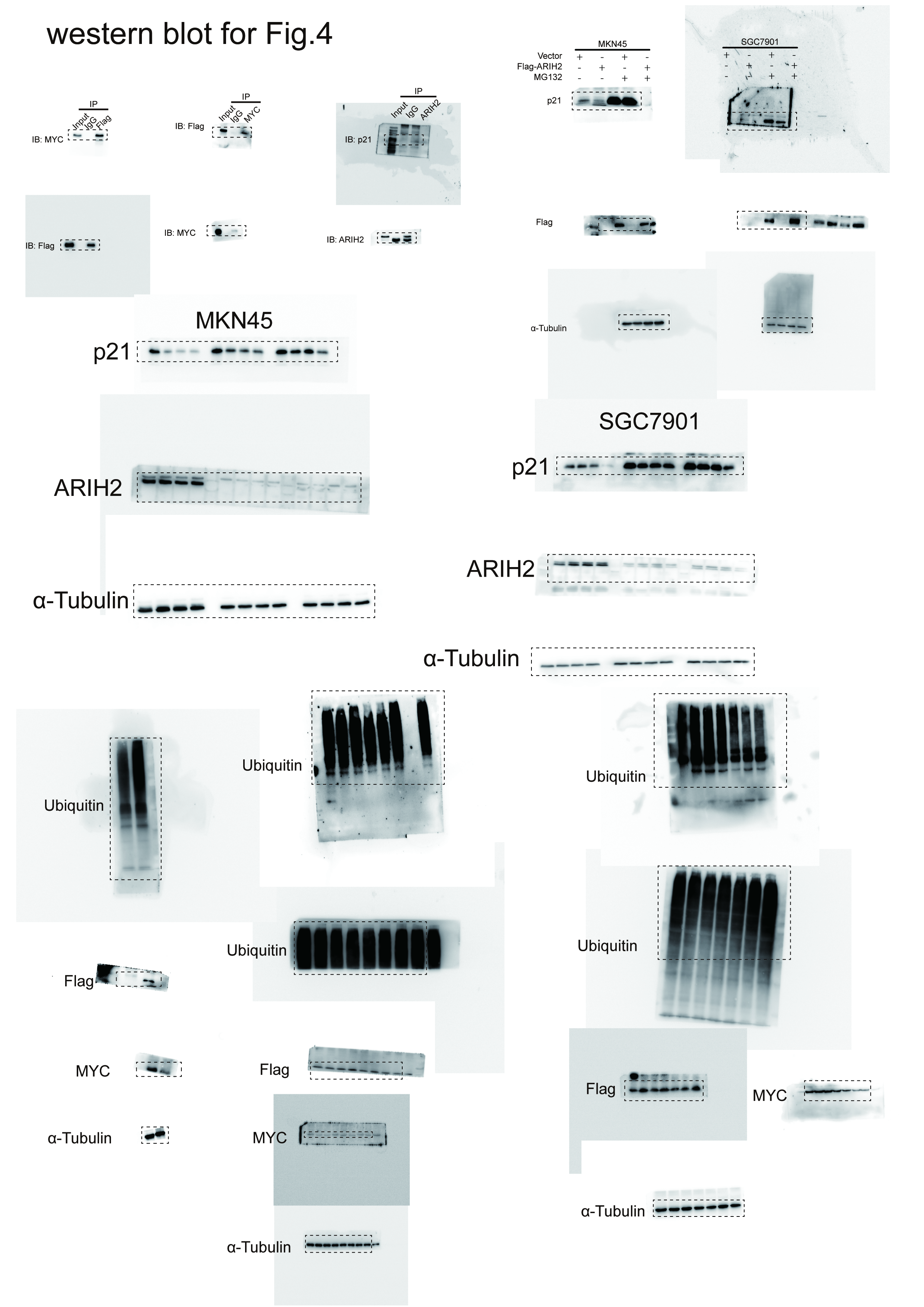

Supplement: Supplementary file 6 — Original Data File [file 41419_2022_4965_MOESM6_ESM.tif]

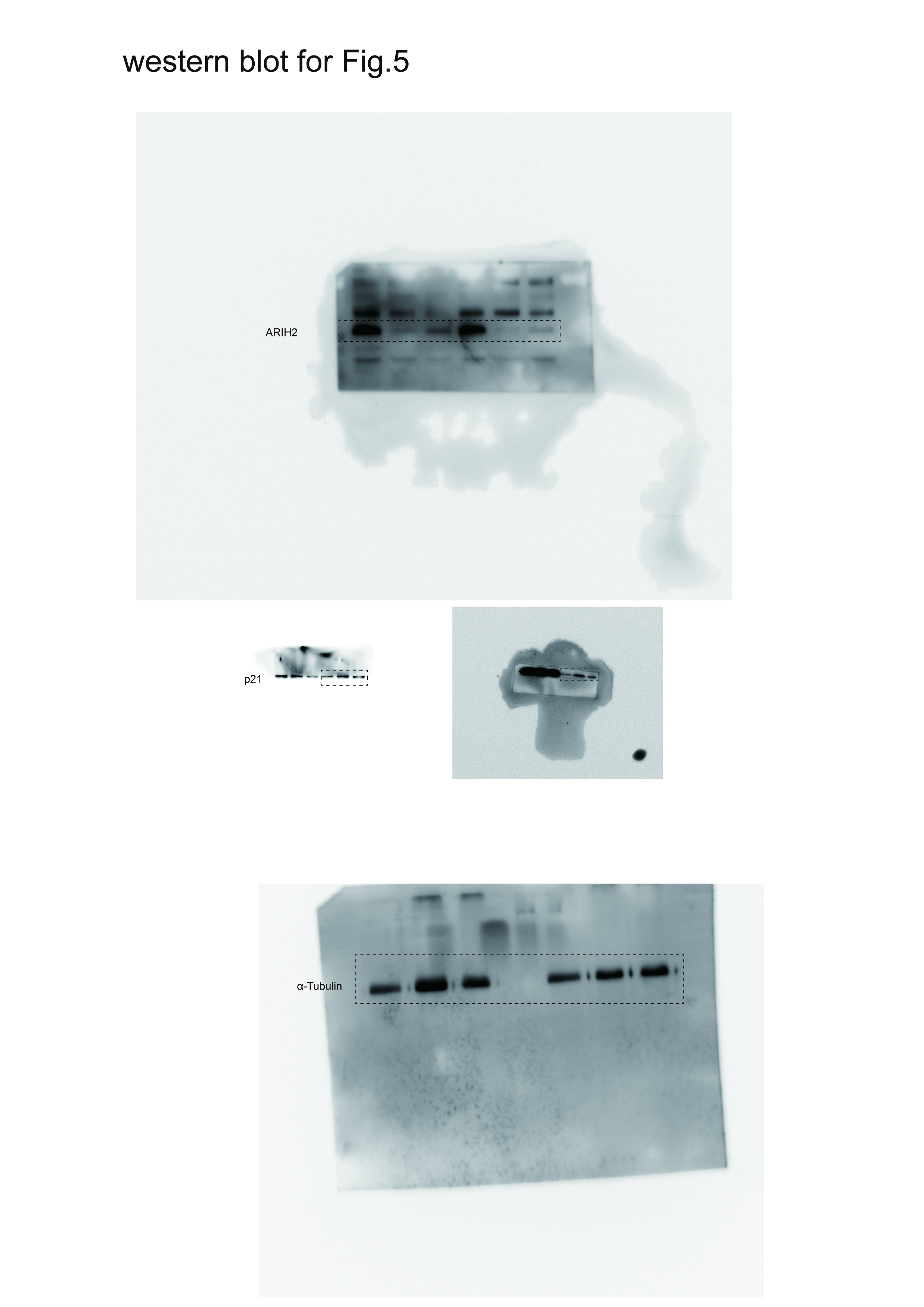

Supplement: Supplementary file 7 — Original Data File [file 41419_2022_4965_MOESM7_ESM.tif]

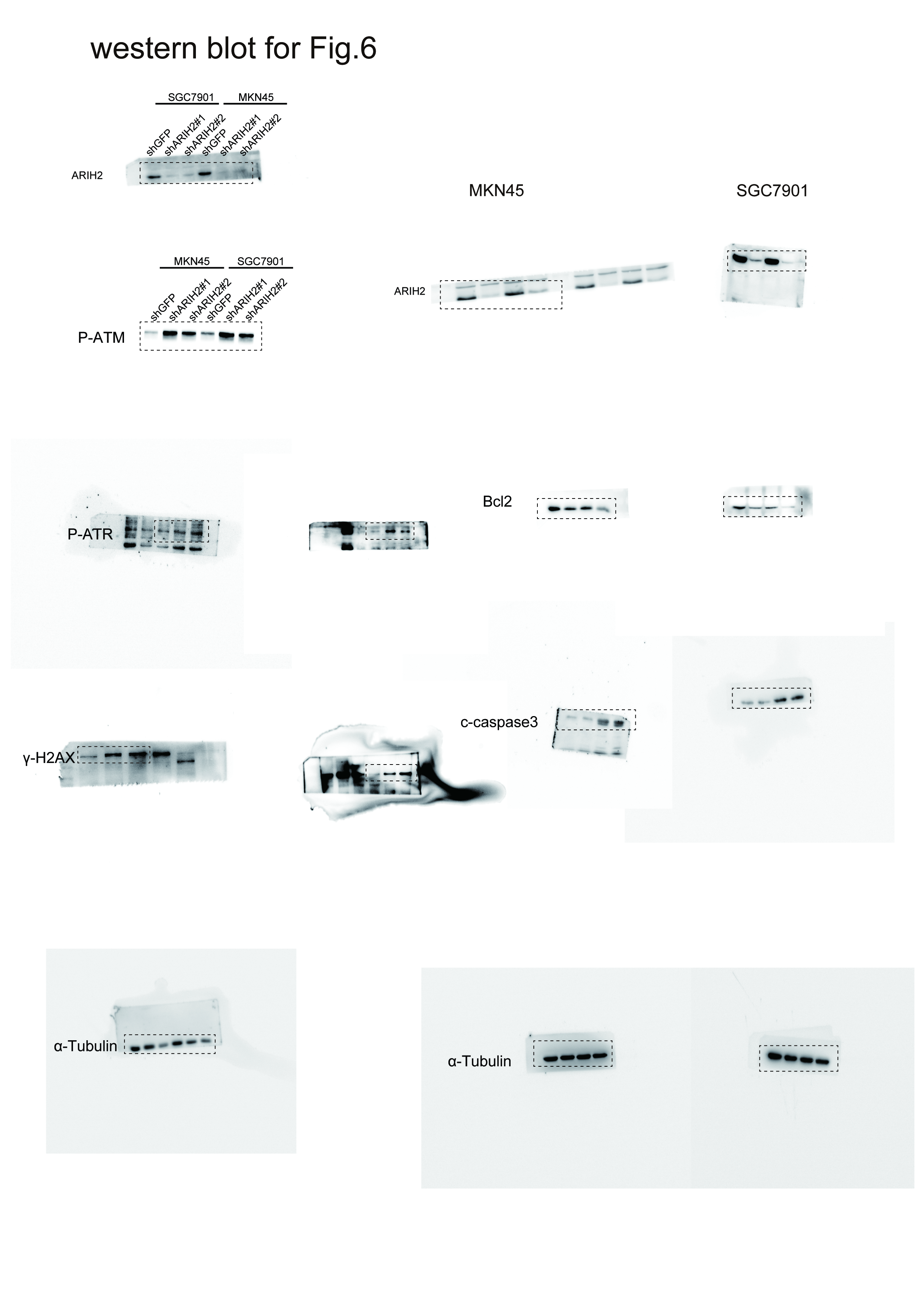

Supplement: Supplementary file 8 — Original Data File [file 41419_2022_4965_MOESM8_ESM.tif]

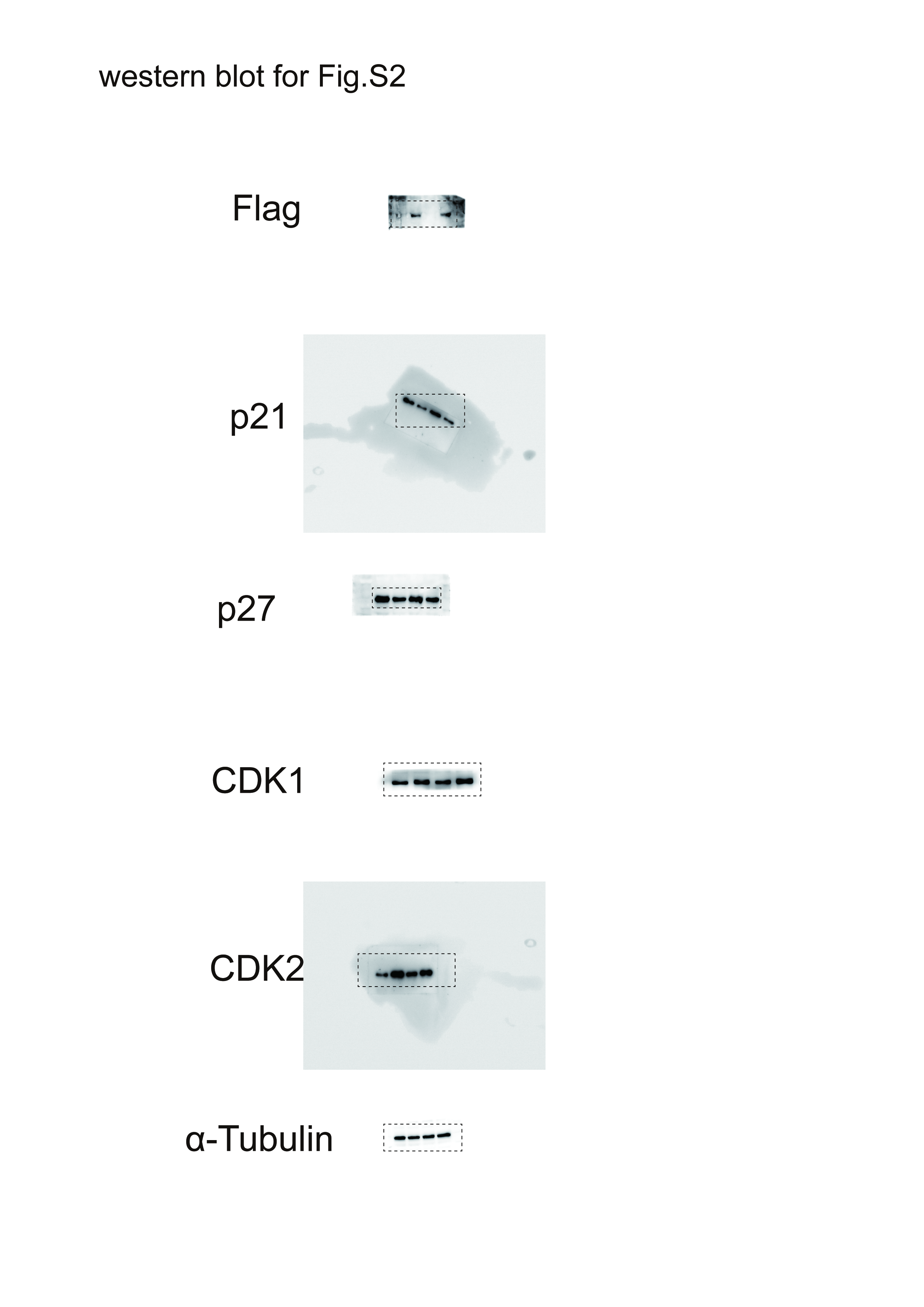

Supplement: Supplementary file 9 — Original Data File [file 41419_2022_4965_MOESM9_ESM.tif]

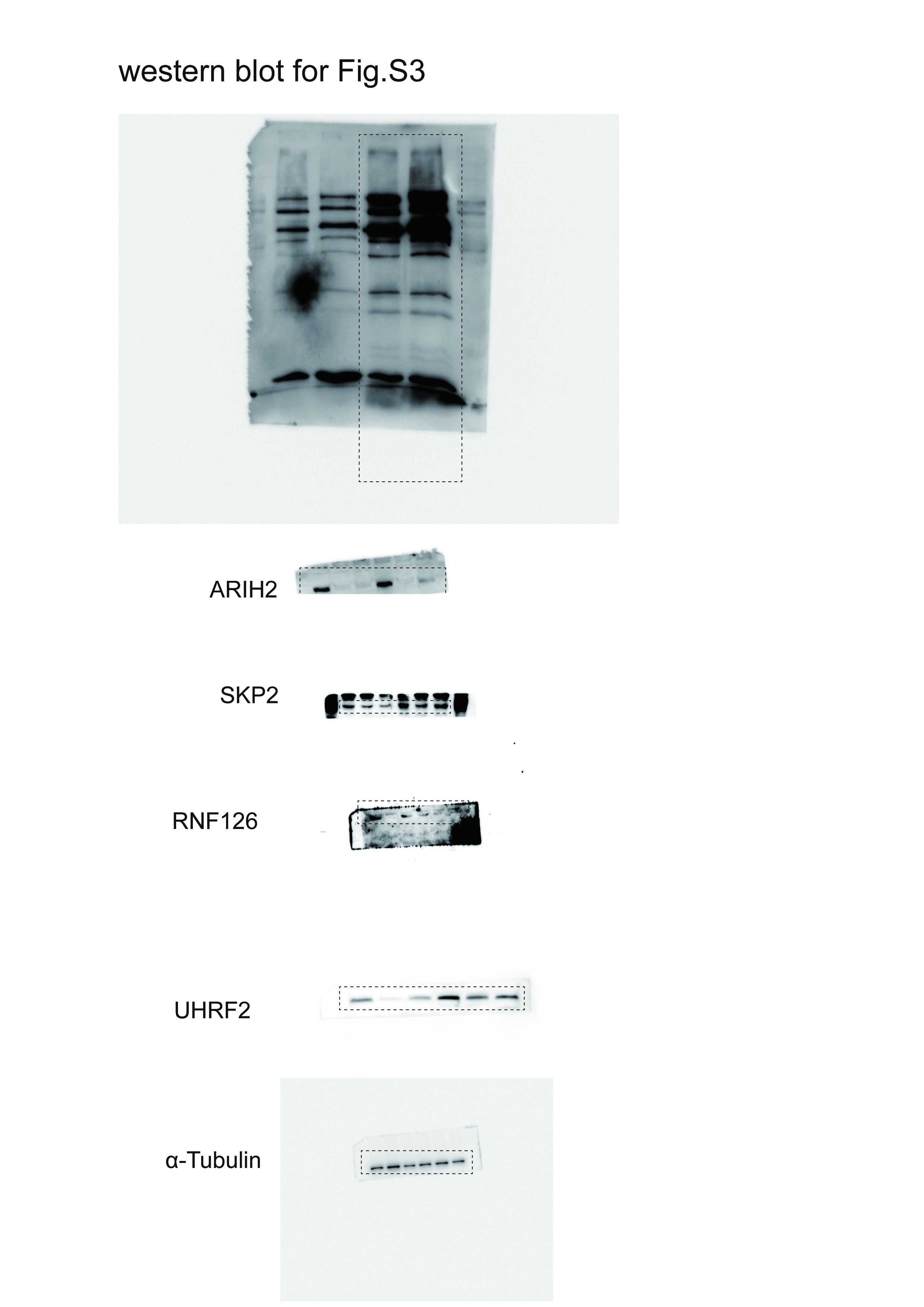

Supplement: Supplementary file 10 — Original Data File [file 41419_2022_4965_MOESM10_ESM.tif]
